# Disconnecting from my heartbeat: About a case of derealization in a critically ill patient

**DOI:** 10.1192/j.eurpsy.2024.869

**Published:** 2024-08-27

**Authors:** C. Alario Ruiz, M. S. Oscar, N. Navarro Barriga, R. R. Beatriz, R. V. Claudia

**Affiliations:** Hospital Clínico Universitario de Valladolid, Valladolid, Spain

## Abstract

**Introduction:**

Depersonalization/derealization encompasses a series of symptoms that are difficult to describe by the patient, as well as complex to diagnose by the professional, and can go through multiple diagnoses prior to the diagnosis of certainty.

**Objectives:**

It is proposed, through a clinical case, to know the characteristics of this disorder, evolution, differential diagnosis and therapeutic possibilities

**Methods:**

62-year-old male, history of harmful alcohol consumption and a previous admission to a psychiatric short hospitalization unit for self-injury (superficial cuts in the context of severe mental illness of his wife) post-transplantation who is required by ideas of death, anhedonia and lack of collaboration in patient, The day before the evaluation, refusal to take oral treatment, selective mutism. Pre-transplant evaluation where no psychopathological alteration was observed.

**Results:**

What is initially assessed and treated as a confusional episode of inactive type, through a correct psychopathological examination and with subsequent continuous interviews, with mood fluctuations throughout the admission, is subsequently oriented as an acute stress disorder, adaptive reaction with an anxious-depressive component and finally concluding that we are facing a dissociative disorder, highlighting the depersonalization/derealization on a dysfunctional personality base.

**Conclusions:**

Characteristic of depersonalization is the great difficulty in describing symptoms, the feeling of being disconnected from one’s own body, emotions and reality. The latest studies on etiopathogenesis with MRI show an inhibitory response on the limbic system by hyperactivation of the ventrolateral prefrontal cortex as well as a decrease in the autonomic response, the initial result being the attenuation of the processing of emotions. Among the differential diagnoses: post-anxiety illness disorder, major depressive episode, other dissociative disorders, panic disorder, psychotic disorder, substance-induced disorder There are several partially effective treatments, although the results so far are poor. SSRIs, quetiapine and naltrexone have been tried. Partial efficacy with lamotrigine together with SSRIs and, if high levels of anxiety coexist, SSRIs together with clonazepam. There are studies where psychodynamic psychotherapy, behavioral therapy and hypnosis have obtained partially effective results.

**Disclosure of Interest:**

None Declared

